# Autonomic mechanisms underpinning the stress response in borderline hypertensive rats

**DOI:** 10.1113/expphysiol.2010.055970

**Published:** 2011-03-18

**Authors:** Olivera Šarenac, Maja Lozić, Srdja Drakulić, Dragana Bajić, Julian F Paton, David Murphy, Nina Japundžić-Žigon

**Affiliations:** 1 Institute of Pharmacology, Clinical Pharmacology and Toxicology, School of Medicine, University of BelgradeRepublic of Serbia; 2 Faculty of Technical Sciences, University of Novi SadRepublic of Serbia; 3 Department of Physiology and Pharmacology, Bristol Heart Institute, School of Medical Sciences, University Walk, University of BristolBristol, UK; 4 The Molecular Neuroendocrinology Research Group, Henry Wellcome Laboratories for Integrative Neuroscience and Endocrinology, University of BristolBristol, UK

## Abstract

This study investigates blood pressure (BP) and heart rate (HR) short-term variability and spontaneous baroreflex functioning in adult borderline hypertensive rats and normotensive control animals kept on normal-salt diet. Arterial pulse pressure was recorded by radio telemetry. Systolic BP, diastolic BP and HR variabilities and baroreflex were assessed by spectral analysis and the sequence method, respectively. In all experimental conditions (baseline and stress), borderline hypertensive rats exhibited higher BP, increased baroreflex sensitivity and resetting, relative to control animals. Acute shaker stress (single exposure to 200 cycles min-1 shaking platform) increased BP in both strains, while chronic shaker stress (3-day exposure to shaking platform) increased systolic BP in borderline hypertensive rats alone. Low- and high-frequency HR variability increased only in control animals in response to acute and chronic shaker (single exposure to restrainer) stress. Acute restraint stress increased BP, HR, low- and high-frequency variability of BP and HR in both strains to a greater extent than acute shaker stress. Only normotensive rats exhibited a reduced ratio of low- to high-frequency HR variability, pointing to domination of vagal cardiac control. In borderline hypertensive rats, but not in control animals, chronic restraint stress (9-day exposure to restrainer) increased low- and high-frequency BP and HR variability and their ratio, indicating a shift towards sympathetic cardiovascular control. It is concluded that maintenance of BP in borderline hypertensive rats in basal conditions and during stress is associated with enhanced baroreflex sensitivity and resetting. Imbalance in sympathovagal control was evident only during exposure of borderline hypertensive rats to stressors.

Mildly elevated blood pressure (BP) or borderline hypertension is a widespread condition in the human population and a major risk factor for developing overt essential hypertension and its complications. Borderline hypertension is a good model to study early autonomic dysregulation of BP because it is devoid of confounding structural changes associated with advanced stages of essential hypertension, particularly arterial wall stiffness that interferes with assessment of baroreflex sensitivity (Larkin 2005).

The baroreflex is the main feedback mechanism regulating BP and is an important contributor to short-term BP and heart rate (HR) variability (Cerutti *et al.* 1994). Impairment of baroreflex function characterizes overt forms of both experimental hypertension (McCubin *et al.* 1956; Nosaka & Omamoto 1970; Matsuguchi & Schmid, 1982; Su *et al.* 1986) and human essential hypertension (Brotman *et al.* 2007; Julien, 2009); however, clinical (Bovy *et al.* 1983; Watkins *et al.* 1996) and experimental (Lawler *et al.* 1991; Sanders & Lawler, 1992) data on baroreflex function in borderline hypertensive subjects are conflicting.

The hallmark of successful treatment of hypertension is the maintenance of the integrity of baroreflex function (Mancia & Parati, 2003; Narkiewicz & Grassi, 2008; Ormezzano *et al.* 2008). Although experimental evidence points to a critical phase when, during the development of hypertension, baroreflex function can be restored with drugs (Kumagai *et al.* 1996), there is still no well-structured algorithm for management and treatment of borderline hypertension (Mancia *et al.* 2009).

As a model of the human condition of borderline hypertension, Lawler and colleagues (Lawler *et al.* 1991; Sanders & Lawler, 1992) developed a rat strain, by cross-breeding the genetically spontaneously hypertensive rat with normotensive rats. The BP of adult borderline hypertensive rats (BHRs) is higher than in normotensive rats and similar to the human borderline condition. In our experiments, the BP of BHRs was 135/99 mmHg, corresponding to high-normal to mild hypertension in humans (Mancia *et al.* 2009). Although BHRs have a genetic predisposition for hypertension, they will not develop overt hypertension spontaneously unless exposed to stress or elevated salt intake (Lawler *et al.* 1991; Sanders & Lawler, 1992), both of which are important risk factors in the pathogenesis of human hypertension (Larkin, 2005; Brotman *et al.* 2007; Julien, 2009). We hypothesized that BHRs have altered neurogenic control of the cardiovascular system, making them vulnerable to environmental stress even in conditions of regular salt intake. To test this hypothesis, we investigated the functioning of the neural cardiovascular control mechanisms of BHRs both in basal physiological conditions and when exposed to stressors eliciting different behavioural and cardiovascular responses. The neural mechanism underlying the cardiovascular response to stressors was assessed by spectral analysis of BP and HR and the sequence method. Preliminary results were presented at the main meeting of the Physiological Society in 2009 (Šarenac *et al.* 2009).

## Methods

All experimental procedures in this study conformed to the European Communities Council Directive of 24 November 1986 (86/609/EEC). The experimental protocol was granted by the School of Medicine, University of Belgrade ethics review board (approval reference number 1306/1–3).

### Animals

Experiments were performed in male, 12-week-old BHRs obtained by cross-breeding Wistar sires with spontaneously hypertensive rat dams purchased from Charles River (Sulzfeld, Germany). Age-matched Wistar rats were used as normotensive control animals. Both strains of rats weighed 340–360 g and were housed individually in a controlled environment: 12 h–12 h light–dark cycle, temperature 21 ± 2°C and humidity 65 ± 9%, with access to standard food pellets (0.2% sodium content; Veterinarski zavod, Subotica, Republic of Serbia) and tap water *ad libitum*. The number of rats in each protocol was calculated statistically, taking into account intragroup variability, using the software package ‘Power Sample Size Calculation’, available at http://biostat.mc.vanderbilt.edu/twiki/bin/view/Main/PowerSampleSize, for power of 90% and type I error probability of 0.05. At the end of the experiment, the rats were killed by an overdose of thiopentone sodium (150 mg, i.p.).

### Surgery

Under combined ketamine (100 mg kg^−1^, i.m.) and xylazine (10 mg kg^−1^, i.m.) general anaesthesia, a 3-cm-long medial abdominal incision was made and the intestine retracted to expose the abdominal aorta. The tip of the catheter of the radio telemetric probe (TA11-PA C40; DSI, Transoma Medical, St Paul, MN, USA) was inserted into the aorta using a 21-gauge needle. The inserted catheter was fixed with 3M Vetbond™ and tissue cellulose patch (DSI, Transoma Medical). The transmitter was attached to the anterior abdominal wall and the wound closed by suturing. To prevent bacterial infection, neomycin and bacitracin were sprayed topically, and the rats were treated with gentamicin (25 mg kg^−1^, i.m.) for 3 days prior to surgery and on the day of surgery. To reduce pain, rats received metamizole (200 mg kg^−1^ day^−1^, i.m.) on the day of surgery and for the next 2 days. Each rat was housed in a Plexiglass cage (30 cm × 30 cm × 30 cm) and left to recover fully for 8–10 days prior to experimentation.

### Experimental protocol

All experiments were started at 10.00 h in a quiet surrounding in controlled environmental conditions. During experimentation, rats were kept in their home cages and randomized into two experimental protocols: shaker stress and restraint stress. The duration of stress depended on the ability of the Wistar rats to adapt and prevent elevation of BP and HR. Adaptation to shaker stress occurred after 3 days and to restraint stress after 9 days.

The shaker stress protocol was therefore performed for 3 days in six rats. Every day, rats were exposed 18 times per day to a shaking platform (200 cycles min^−1^) for 10 min, starting at 08.00 h and finishing at 20.00 h. Periods between shaking episodes were variable (15, 30 or 45 min) and were selected randomly. On the first day of the study, BP was recorded for 20 min before the first exposure to stress (baseline) and 10 min during the first exposure (acute shaker stress). On day 3, BP was recorded 10 min during the last exposure to stress (chronic shaker stress) and 20 min after the last exposure to shaker stress.

The restraint stress protocol was performed for 9 days in six rats. Every day, rats were placed in a Plexiglass tube (internal diameter 5.5 cm, with ventilation pores) in the supine position for 1 h. On the first day of experimentation, BP was recorded 20 min before stress (baseline) and 60 min during the first exposure to stress (acute restraint stress). On day 9, BP was recorded 60 min during the last exposure to stress (chronic restraint stress) and 20 min after the last exposure to restraint stress.

### Cardiovascular signal processing and analysis

Arterial blood pressure was digitalized at 1000 Hz using Dataquest A.R.T. 4.0 software, (DSI, Transoma Medical). Systolic and diastolic BP and pulse interval (PI) or its inverse, HR, were derived from the arterial pulse pressure as maximum, minimum and interbeat interval of the pulse pressure wave, respectively.

### Evaluation of the spontaneous baroreceptor reflex by the method of sequences

**Figure 1:**
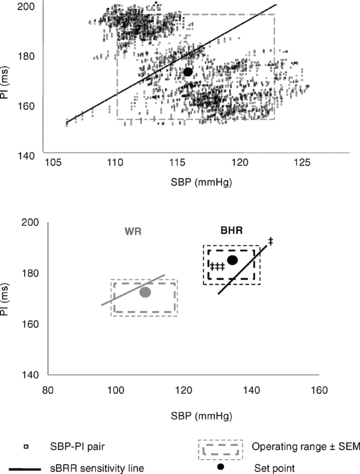
Features of the spontaneous baroreflex in baseline conditions in borderline hypertensive rats (BHRs) and Wistar rats Upper panel illustrates baroreflex sensitivity (line) in one Wistar rat calculated as the linear regression coefficient of the sequence averaged over all identified sequences, operating range (dashed rectangle) covering 95% of systolic blood pressure (BP)–pulse interval (PI) pairs that form sequences, and set point (filled circle) calculated as median value of all set points. Lower panel shows mean baroreflex sensitivity, operating range ± SEM and set point for 12 Wistar rats (WR; grey) and 12 BHRs (black). Note the resetting and the increase in baroreflex sensitivity of BHRs. ‡*P* < 0.05, ‡‡‡*P* < 0.001 BHRs *versus* WRs.

The method is explained in detail elsewhere (Bajić *et al.* 2010). Briefly, a spontaneous baroreflex sequence is a stream of consecutively increasing/decreasing systolic BP samples, followed by a stream of increasing/decreasing PI interval samples delayed by three, four or five beats with respect to BP. A threshold for sequence length was set to four beats (Lončar-Turukalo *et al.* 2011). Sequences were identified on 7-min-long registration periods, and the following baroreflex features were evaluated (Fig. 1):

- sensitivity (in ms mmHg^−1^), assessed as a linear regression coefficient averaged over all identified sequences (pulse interval = baroreflex sensitivity × systolic BP + constant, where fitting of the curve is done in a least-squares sense);
- effectiveness index, calculated as the ratio of the number of sequences to the number of systolic BP ramps;
- operating range (in ms mmHg) is obtained from the rectangle surface covering 95% of systolic BP–PI pairs that form sequences, in both dimensions of the systolic BP and PI plane; and
- set point for PI (in ms) and for systolic BP (in mmHg) is calculated as the median value of all systolic BP–PI sequence points.

### Spectral analysis of BP and HR

Before spectral analysis was performed, systolic and diastolic BP and HR signals were resampled at 20 Hz and subjected to nine-point Hanning window filter and linear trend removal, detailed elsewhere (Japundžić-Žigon, 2001). Spectra were obtained using a fast Fourier transform (FFT) algorithm on 15 overlapping 2048-point time series corresponding to a 410 s (∼7 min) registration period of systolic and diastolic BP and HR. The power spectrum of BP (in mmHg^2^) and HR [in (beats min^-1^)^2^] for 30 FFT segments was calculated for the whole spectrum (total, 0.0195–3 Hz) and in the following three frequency ranges: very low frequency (VLF, 0.0195–0.195 Hz), low frequency (LF, 0.195–0.8 Hz) and high frequency (HF, 0.8–3 Hz). In order to assess spectral frequency distribution, BP and HR spectra were expressed in normalized units (frequency range/total spectral power). The LF oscillation of systolic and diastolic BP spectrum (LF SBP and LF DBP) and LF/HF HR are recognized clinical markers of sympathetic modulation of vascular tone and sympathovagal balance to the heart, respectively (Parati *et al.* 1995; Task Force, 1996).

### Drugs

Ketamine and xylazine were purchased from Richter Pharma (Wels, Austria) and Céva Santé Animal (Budapest, Hungary), respectively. Metamizol and gentamicin injections were purchased from Hemofarm (Vršac, Republic of Serbia) and Enbencin® spray (neomycin plus bacitracin) from Galenika (Belgrade, Republic of Serbia).

### Statistics

Cardiovascular parameters are shown as means ± SEM. Cardiovascular responses of different rat strains submitted to the same experimental protocol were compared by two-way ANOVA for repeated measures followed by *post hoc* Bonferroni test using GraphPad Prism 4 software (GraphPad Software Inc., San Diego, CA, USA). Comparisons between stressors within the same strain were made by Student's *t* test for unpaired observations. Statistical significance was considered at *P* < 0.05.

## Results

### Cardiovascular parameters in BHRs and Wistar rats in baseline conditions

**Figure 2:**
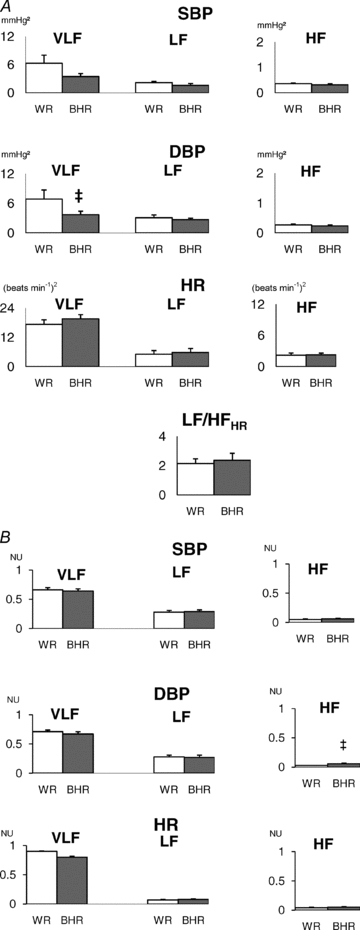
Blood pressure and heart rate spectral components in BHRs and Wistar rats in baseline conditions In this and the following figures, abbreviations are as follows: BHR, borderline hypertensive rat; BP, blood pressure; DBP, diastolic BP; HF, high frequency spectral power; HR, heart rate; LF, low frequency spectral power; SBP, systolic BP; TOTAL, total spectral power; VLF, very low frequency spectral power; and WR, Wistar rat. *A* (absolute values), note the decrease of VLF variability in diastolic BP spectra in BHRs. *B* (normalized units), note redistribution of spectral frequencies towards HF variability in diastolic BP spectrum of BHRs. Values are means of 12 rats ± SEM. ‡*P* < 0.05 BHRs *versus* WRs.

In baseline conditions, BHRs had significantly higher systolic and diastolic BP than Wistar rats, while the HR was not significantly different (Table 1). The sequence method revealed that the baroreflex of BHRs was reset towards higher systolic BP values (Fig. 1), and worked with enhanced sensitivity (Fig. 1 and Table 1) in comparison with Wistar rats. Analysis of the HR and the BP spectra showed no difference between Wistar rats and BHRs in any spectral parameter, i.e. LF SBP, LF HR and LF/HF HR. Only in diastolic BP spectra, a decrease of VLF variability was noted in BHRs in comparison with Wistar rats (Fig. 2*A*). Normalized BP spectra indicated redistribution of spectral frequencies towards the HF range, as indicated by the increase in comparison with Wistar rats (Fig. 2*B*).

**Table 1:** Blood pressure, heart rate and baroreflex sensitivity of borderline hypertensive rats (BHRs) and Wistar rats in baseline conditions

	Wistar rats	BHRs
SBP (mmHg)	109 ± 3.0	134 ± 3.7‡‡‡
DBP (mmHg)	79 ± 3.0	99 ± 3.4‡‡‡
HR (beats min^-1^)	352 ± 7.8	328 ± 10.1
BRS (ms mmHg^−1^)	1.5 ± 0.2	2.2 ± 0.3‡

Values are means of 12 rats ± SEM. Abbreviations: BRS, baroreflex sensitivity; DBP, diastolic blood pressure; HR, heart rate; SBP, systolic blood pressure.

‡ *P* < 0.05

‡‡‡ *P* < 0.001 BHRs *versus* Wistar rats.

### Cardiovascular response of BHRs and Wistar rats to acute shaker stress

**Figure 3:**
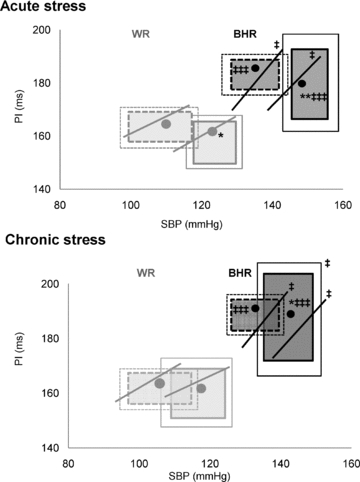
Baroreflex parameters in BHRs and Wistar rats exposed to shaker stress Acute stress induces resetting of the baroreflex in both rat strains. Chronic shaker stress resets the baroreflex only in BHRs. Baseline, dashed line; stress, continuous line. Values are means of six rats ± SEM (dotted line). *P < 0.05, **P < 0.01 stress *versus* baseline; ‡*P* < 0.05, ‡‡‡*P* < 0.001 BHRs *versus* WRs.

**Figure 4:**
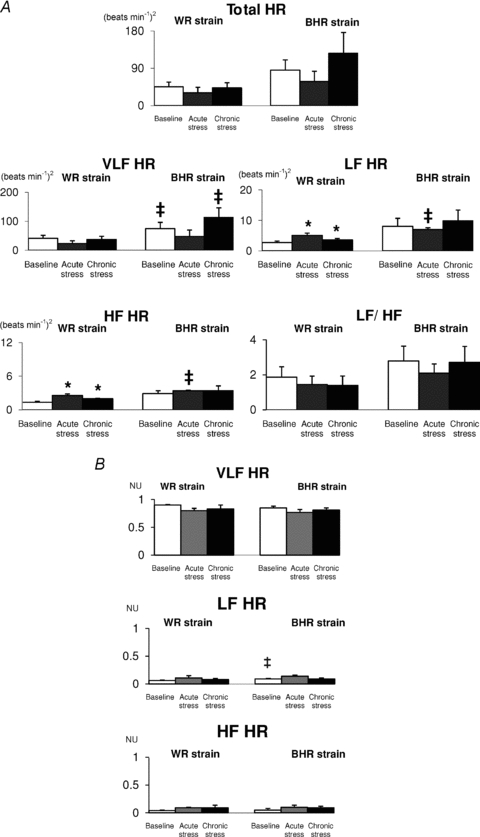
Effects of shaker stress on HR spectral components in BHRs and Wistar rats *A* (absolute values), acute and chronic stress increases LF and HF HR variability only in Wistar rats, without affecting LF/HF HR ratio. *B* (normalized units), exposure of BHRs and Wistar rats to acute and chronic stress did not induce redistribution of HR spectral frequencies. Values are means of six rats ± SEM. *P < 0.05 stress *versus* baseline; ‡*P* < 0.05 BHRs *versus* WRs.

Shaker stress induced a characteristic behavioural response in both rat strains. During the first exposure to stress, all rats exhibited a freezing reaction, i.e. total absence of spontaneous motor activity, and remained in a balancing posture (extended legs). Cardiovascular changes in Wistar rats were characterized by a significant increase of systolic and diastolic BP (Table 2 and Fig. S1). The baroreflex of Wistar rats was reset towards higher systolic BP values (Fig. 3) and exhibited same sensitivity (Table 2), effectiveness index (0.9 ± 01 baseline *versus* 0.8 ± 0.02 acute stress; *P* > 0.05) and operating range as in baseline conditions (Fig. 3). Heart rate spectral changes of Wistar rats were characterized by increased LF and HF variability without changes in the LF to HF ratio (Fig. 4*A* and Fig. S1) or redistribution of spectral frequencies in normalized HR spectra (Fig. 4*B*). This finding reflects unchanged cardiac sympathovagal balance.

**Table 2:** Blood pressure, heart rate and baroreflex sensitivity of BHRs and Wistar rats exposed to shaker stress

	Wistar rats			Borderline hypertensive rats		
		
	Baseline	Acute stress	Chronic tress	Baseline	Acute stress	Chronic stress
SBP (mmHg)	111 ± 2.8	125 ± 4.5**	119 ± 1.7	136 ± 2.1‡‡‡	149 ± 3.4**‡‡‡	143 ± 4.0*‡‡‡
DBP (mmHg)	79 ± 2.9	95 ± 2.0*	90 ± 3.0	99. ± 3.2‡‡‡	110 ± 4.0*‡	105 ± 3.9‡
HR (beats min^−1^)	351 ± 8.2	384 ± 11.5	403 ± 38.8	327 ± 9.1	338 ± 14.2‡	333 ± 23.5‡
BRS (ms mmHg^−1^)	1.5 ± 0.2	1.0 ± 0.3	1.3 ± 0.3	2.5 ± 0.6‡	1.8 ± 0.2‡	2.1 ± 0.5‡

Values are means of six rats ± SEM. Abbreviations are as in Table 1.

* *P* < 0.05

** *P* < 0.01 baseline *versus* stress

‡ *P* < 0.05

‡‡‡ *P* < 0.001 BHRs *versus* Wistar rats.

**Figure 5:**
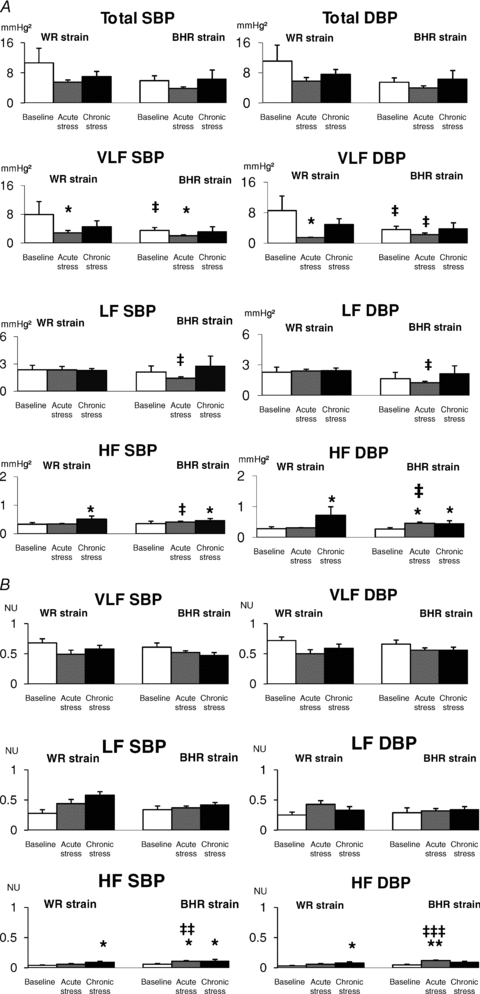
Effects of shaker stress on BP spectral components in BHRs and Wistar rats *A* (absolute values), in both strains chronic shaker stress increased respiration-induced HF systolic and diastolic BP variability. *B* (normalized units), note a shift of spectral frequencies towards the HF band in both systolic and diastolic BP spectra of both rat strains exposed to shaker stress. Values are means of six rats ± SEM. *P < 0.05, **P < 0.01 stress *versus* baseline; ‡*P* < 0.05, ‡‡*P* < 0.01, ‡‡‡*P* < 0.001 BHRs *versus* WRs.

Borderline hypertensive rats exposed to acute stress exhibited a greater increase in maximal systolic and diastolic BP, while the difference of increase in diastolic BP was smaller than in Wistar rats (Table 2 and Fig. S2). No change in HR, compared with baseline conditions, occurred (Table 2). The baroreflex of BHRs was reset towards higher systolic BP and lower PI values; effectiveness index (0.8 ± 0.02 baseline *versus* 0.76 ± 0.04 in acute stress; *P* > 0.05) and operating range remained unchanged (Fig. 3), while baroreflex sensitivity was enhanced compared with Wistar rats (Table 2 and Fig. 3). Spectral analysis of HR (Fig. 4*A* and *B* and Fig. S2) revealed no changes relative to baseline conditions. In the systolic BP spectrum, VLF was decreased, while in the systolic and diastolic BP spectra, HF was increased (Fig. 5*A* and *B*).

### Cardiovascular response of BHRs and Wistar rats to chronic shaker stress

Repeated exposure to stress reduced spontaneous motor activity in all rats, which remained in a typical balancing posture (with extended legs) throughout the shaking episode. Systolic and diastolic BP, HR (Table 2) and baroreflex parameters of Wistar rats remained unaffected (Fig. 3), while LF and HF heart rate variability still increased without a change in LF to HF heart rate ratio (Fig. 4*A*) or redistribution of frequencies (Fig. 4*B*). In addition, an increase in the respiratory, HF BP short-term variability was observed (Fig. 5*A* and *B*). This effect was probably due to stress-induced changes in breathing pattern, i.e. an increase in the depth of breathing. The frequency of breathing, before stress and during exposure to stress, did not change, as evaluated by the position of the HF peak in the BP spectrum (1.32 ± 0.16 Hz before stress *versus* 1.33 ± 0.24 Hz during exposure to stress; *P* > 0.05).

In BHRs exposed to chronic stress, systolic BP still increased significantly (Table 2), and the baroreflex was reset towards higher systolic BP values, and worked with enhanced sensitivity and operating range compared with Wistar rats (Fig. 3 and Table 2). In the spectra of BP (Fig. 5*A* and *B*) and HR (Fig. 4*A* and *B*) of BHRs, there were no changes relative to baseline conditions except for the increase of respiration-induced HF BP variability (Fig. 5*A* and *B*).

### Cardiovascular response of BHRs and Wistar rats to acute restraint stress

**Figure 6:**
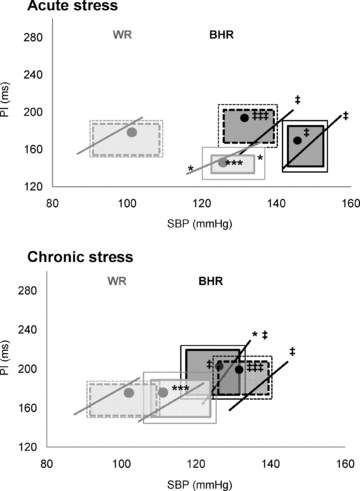
Baroreflex parameters in BHRs and Wistar rats exposed to restraint stress Acute stress resets the baroreflex of Wistar rats and reduces its sensitivity and operating range. Chronic stress increases the sensitivity of BHRs with respect to baseline values and Wistar rats. Baseline, dashed line; stress, continuous line. Values are means of six rats ± SEM (dotted line). *P < 0.05, ***P < 0.001 stress *versus* baseline; ‡*P* < 0.05, ‡‡‡*P* < 0.001 BHRs *versus* WRs.

**Figure 7:**
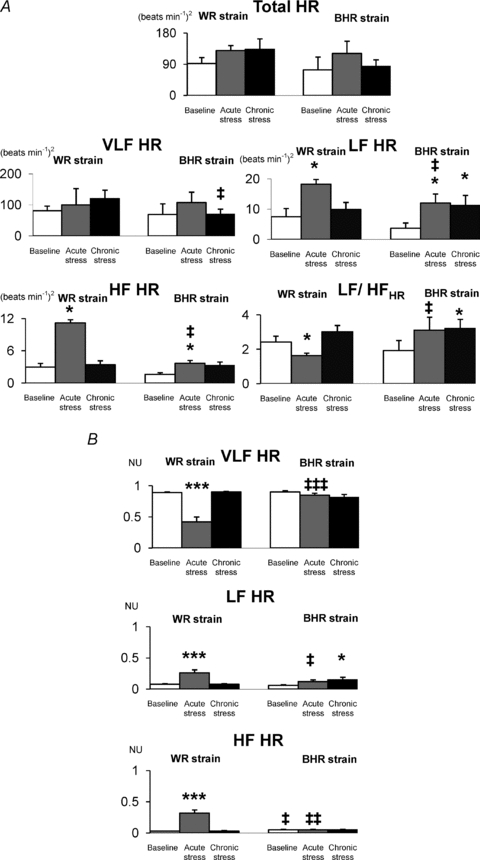
Effects of restraint stress on HR spectral components of BHR and Wistar rats *A* (absolute values), in both strains of rats acute stress increases LF and HF HR variability. Only in Wistar rats the LF to HF ratio decreases, pointing to domination of vagal cardiac control. Chronic exposure to stress increases LF HR variability and LF to HF HR ratio only in BHRs, indicating a shift towards sympathetic cardiac control. *B* (normalized units), acute stress induces clustering of spectral frequencies around LF and HF only in Wistar rats. Chronic stress increases LF frequency only in BHRs. Values are means of six rats ± SEM. *P < 0.05, ***P < 0.001 stress *versus* baseline; ‡*P* < 0.05, ‡‡*P* < 0.01, ‡‡‡*P* < 0.001 BHRs *versus* WRs.

**Figure 8:**
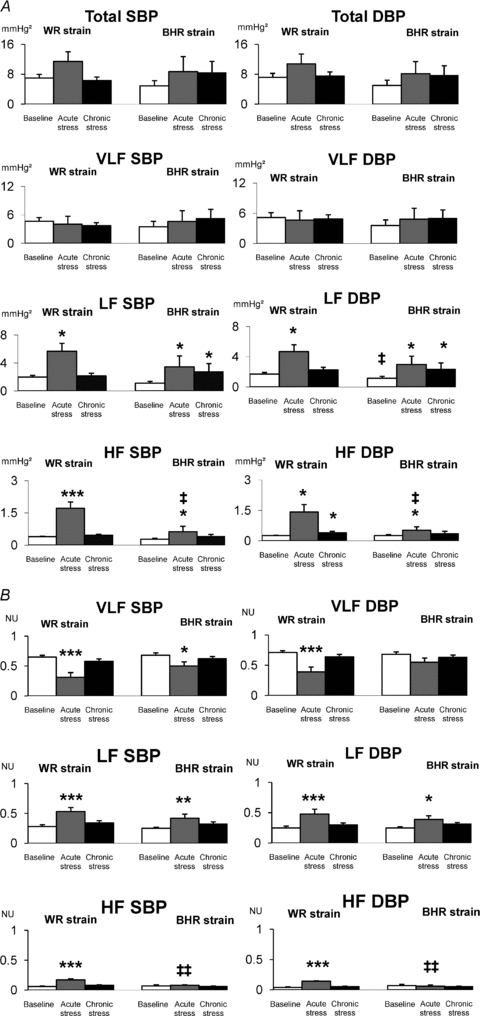
Effects of restraint stress on BP spectral components of BHRs and Wistar rats *A* (absolute values), acute stress increases LF and HF systolic and diastolic BP variability in both strains of rats. Chronic exposure to stress increases LF systolic and diastolic BP variability only in BHRs, indicating an increase of sympathetic outflow to resistance blood vessels. *B* (normalized units), acute stress redistributes spectral frequencies around LF and HF systolic and diastolic BP variability in Wistar rats, while in BHRs the frequencies are clustered around LF variability alone. Values are means of six rats ± SEM. *P < 0.05, **P < 0.01, ***P < 0.001 stress *versus* baseline; ‡*P* < 0.05, ‡‡*P* < 0.01 BHRs *versus* WRs.

Rats of both strains put in restraining tubes struggled to escape. In Wistar rats exposed to acute restraint stress, systolic and diastolic BP and HR increased (Table 3 and Fig. S1). The baroreflex was reset and functioned with reduced sensitivity (Table 3) over a smaller operating range compared with baseline conditions (Fig. 6). The effectiveness index remained unchanged (0.8 ± 0.03 baseline *versus* 0.8 ± 0.1 acute restraint; *P* > 0.05). Spectral analysis of HR variability of Wistar rats exposed to acute restraint revealed a LF and HF increase (Fig. 7*A* and *B* and Fig. S1) and reduction of the LF to HF HR ratio compared with baseline conditions (Fig. 7*A*). This indicates that the sympathovagal balance at the heart is shifted towards vagal control in respect to baseline conditions. In addition, LF and HF systolic and diastolic BP spectral components increased with respect to baseline (Fig. 8*A* and *B* and Fig. S1). Acute restraint stress increased the HR of Wistar rats more than acute shaker stress (384 ± 12 *versus* 454 ± 9 beats min^-1^; *P* < 0.001), as well as BP variability (LF SBP was 5.7 ± 1.1 mmHg^2^ during restraint *versus* 2.4 ± 0.4 mmHg^2^ during shaker stress, *P* < 0.05; LF DBP was 4.7 ± 0.9 mmHg^2^ during restraint *versus* 2.4 ± 0.4 mmHg^2^ during shaker stress, *P* < 0.05; HF SBP was 1.7 ± 0.3 mmHg^2^ during restraint stress *versus* 0.3 ± 0.02 mmHg^2^ during shaker stress, *P* < 0.001; and HF DBP was 1.4 ± 0.4 mmHg^2^ during restraint *versus* 0.4 ± 0.04 mmHg^2^ during shaker stress, *P* < 0.01).

**Table 3:** Blood pressure, heart rate and baroreflex sensitivity of BHRs and Wistar rats exposed to restraint stress

	Wistar rats			Borderline hypertensive rats		
		
	Baseline	Acute stress	Chronic stress	Baseline	Acute stress	Chronic stress
SBP (mmHg)	110 ± 2.1	123 ± 4.6**	112 ± 4.7	135 ± 4.0‡‡‡	146 ± 6.5*‡‡‡	132 ± 6.3‡‡‡
DBP (mmHg)	79 ± 3.2	91 ± 5.0***	80 ± 5.2	99 ± 2.4‡‡‡	111 ± 5.8*‡	93 ± 4.7‡
HR (beats min^−1^)	351 ± 5.9	454 ± 9.2***	362 ± 9.4	328 ± 9.8	377 ± 25.6*‡	322 ± 17.4‡
BRS (ms mmHg^−1^)	1.5 ± 0.1	0.5 ± 0.2***	1.4 ± 0.2	2.1 ± 0.1‡	2.1 ± 0.3‡	4.1 ± 1.9*‡

Values are means of six rats ± SEM. Abbreviations are as in Table 1.

* *P* < 0.05

** *P* < 0.01

*** *P* < 0.001 stress *versus* baseline

‡ *P* < 0.05

‡‡‡ *P* < 0.001 BHRs *versus* Wistar rats.

In BHRs, acute restraint stress increased systolic BP, diastolic BP and HR with respect to baseline (Table 3 and Fig. S2). The baroreflex of BHRs was reset towards higher BP and lower PI values (Fig. 6) and was functioning with enhanced sensitivity compared with Wistar rats (Table 3). Both LF and HF components of the systolic and diastolic BP spectrum (Fig. 8*A* and *B* and Fig. S2) and the HR spectrum increased (Fig. 7*A* and *B* and Fig. S2). No change in LF/HF HR ratio was observed (Fig. 7*A* and *B*). Normalized spectra of BP confirmed the redistribution of spectral frequencies towards the LF band (Fig. 8*B*).

### Cardiovascular response of BHRs and Wistar rats to chronic restraint stress

Repeatedly restrained rats of both strains showed lower levels of struggling than during acutely restraint. In Wistar rats exposed to chronic restraint stress, there was no change in BP, HR, baroreflex sensitivity (Table 3), baroreflex effectiveness index (0.8 ± 0.03 baseline *versus* 0.8 ± 0.02 in chronic stress; *P* > 0.05), operating range (Fig. 6), BP short-term variability (Fig. 8*A* and *B*) and HR short-term variability (Fig. 7*A* and *B*). Only the baroreflex set point was still shifted towards slightly higher BP values compared with baseline (Fig. 6). Chronic restraint increased LF HR variability [9.9 ± 2.4 *versus* 3.3 ± 1.5 (beats min^-1^)^2^; *P* < 0.05] and LF/HF HR ratio (2.9 ± 0.5 *versus* 1.5 ± 0.5; *P* < 0.001) more than chronic shaker stress.

The BP and HR of BHRs exposed to chronic restraint returned to baseline. While the BP of BHRs remained higher than the BP of Wistar rats, the HR was significantly lower. The baroreflex of BHRs worked with enhanced sensitivity compared with normotensive rats and with baseline (Table 3 and Fig. 6). Chronic restraint increased the LF spectral component of systolic and diastolic BP of BHRs (Fig. 8*A* and *B*). Also, the LF component of the HR spectrum and the LF/HF HR ratio increased with respect to baseline (Fig. 7*A*). The normalized HR spectrum confirmed the redistribution of frequencies towards the LF band (Fig. 7*B*), pointing to the domination of sympathetic control of the heart and the blood vessels.

In both strains of rats, cardiovascular parameters normalized immediately in the poststress period and are not shown because they do not differ from baseline values.

## Discussion

This study shows that BHRs have higher BP than Wistar rats and that the maintenance of BP in BHRs is associated with enhanced sensitivity and resetting of the baroreflex. The BP of BHRs in our experiments is comparable to the BP of BHRs reported by others (Sanders *et al.* 1988; Lawler *et al.* 1991); however, others reported no statistically significant difference between BP of normotensive rats and BHRs. This discrepancy may be due to different methods of BP assessment or differences in neuroendocrine and emotional traits in different normotensive rat strains (Okamoto & Aoki, 1963; Paré, 1989; Sands *et al.* 2000). The BHR is a well-recognized animal model for studying environmental influences on the development of neurogenic essential hypertension (Sanders *et al.* 1988; Lawler *et al.* 1991; Sanders & Lawler, 1992; Fuchs *et al.* 1998; Di Bona & Jones, 1991*b*; Larkin, 2005). Lawler *et al.* (1991) exposed BHRs for several weeks to tail shock and investigated baroreflex sensitivity using vasoactive drugs. They found that 5 weeks of exposure to this stressor does not alter reflex gain, but resets the baroreflex to a higher BP range. Indeed, 11 weeks of stress was required before baroreflex sensitivity decreased, and this was associated with the development of overt hypertension. In contrast, Gelsema *et al*. (1994) and Lemaire & Mormède (1995) exposed BHRs to chronic social stress for 6 weeks, and failed to induce hypertension.

We used the sequence methodology to evaluate the functioning of the spontaneous baroreflex. This method is reliable both in animals and in humans (Bertinieri *et al.* 1988; Parlow *et al.* 1995; Oosting *et al.* 1997) and does not require the use of vasoactive drugs that interfere with the analysis of the reflex responses (Bertinieri *et al.* 1988; Oosting *et al.* 1997; Casadei & Paterson, 2000; Paton *et al.* 2001). Our findings indicate that the baroreflex of BHRs exposed to stress is reset towards higher BP values and that it works with the same sensitivity as in baseline conditions. However, comparison with normotensive rats revealed that the sensitivity of the baroreflex of BHRs is enhanced both in baseline and in stress conditions. Thus, our study complements and extends the original findings of Lawler *et al.* (1991). Our results might also provide an explanation for the findings of Gelsema *et al.* (1994) and Lemaire & Mormède (1995) in BHRs.

Spectral analysis of BP in BHRs in baseline conditions indicates that diastolic BP variability is reduced in the VLF domain, relative to normotensive rats. The VLF variability dominates BP spectra in baseline physiological conditions (Akselrod *et al.* 1985; Japundžić-Žigon *et al.* 1990; Janssen *et al.* 1995). Its origins are complex, and have been found to arise from the dynamic interaction of the spontaneous blood vessel wall muscle activity in mesenteric and renal vascular beds (Janssen *et al.* 1995) and vasoactive hormones involved in the regulation of local blood flow (Japundžić-Žigon *et al.* 1990; Grichois *et al.* 1992; Ponchon & Elghozi, 1996; Blanc *et al.* 1999). The VLF variability of BP can also comprise thermoregulation-induced changes of the vascular resistance (Milutinović *et al.* 2006). Normally, VLF BP variability is dampened by the baroreflex, as evidenced by debuffering in open-loop experiments in rats (Cerutti *et al.* 1994). Hence, a reduction of VLF variability of BHRs in baseline conditions could be a consequence of the enhanced baroreflex sensitivity and more efficient dampening of diastolic BP. The pathophysiological meaning of the present findings is that the baroreflex of BHRs is reset towards higher BP values but works more efficiently to keep BP within a homeostatic range. Enhanced baroreflex sensitivity could be elicited by inherently greater cardiovascular responsiveness of BHRs to environmental challenges (Sanders & Lawler, 1992) or perhaps is an independent trait. Over time, working with enhanced sensitivity could lead to the breakdown of the baroreflex mechanism and overt hypertension, as reported by Lawler *et al*. (1991).

Enhanced cardiovascular responsiveness of the BHR to environmental challenges has been largely documented; BHRs exposed to heterotypic stressors, painful stimuli (foot shock or tail shock), sodium overload with or without air-jet stress or restraint (Sanders *et al.* 1988; Sanders & Lawler, 1992; Fuchs *et al.* 1998) exhibit a greater increase of one or more haemodynamic parameters (BP, HR, total peripheral resistance and cardiac output) along with plasma catecholamine concentration, in comparison with normotensive rats. Enhanced sympathoadrenal response (Sanders *et al.* 1988; Sanders & Lawler, 1992; Fuchs *et al.* 1998), sensitization of sympathetic response (Mansi & Drolet, 1997), salt-dependent enhancement of sympathetic to vascular signalling (Brown *et al.* 1999), as well as decreased vascular endothelium-dependent relaxation (Fuchs *et al.* 1998; Giulumian *et al.* 1999; Bernatova & Csizmadiova, 2006) were mentioned as peripheral mechanisms. Central α_2_-adrenergic receptors (Di Bona & Jones, 1991*b*) and the anteroventral third ventricle region (Hatton *et al.* 1991) were suggested as the source of increased central sympathetic drive that underlies cardiovascular hyper-responsiveness to environmental challenges (Di Bona & Jones, 1991*a*; Sanders & Lawler, 1992). However, others failed to record enhanced sympathoadrenal and catecholamine responsiveness to environmental challenges in the BHR (Kirby *et al.* 1989; Stratton *et al.* 1994), so the mechanisms that underlie cardiovascular hyper-responsiveness of BHRs to environmental challenges still remain controversial.

To assess the contribution of the autonomic nervous system in cardiovascular regulation, we used spectral analysis of BP and HR short-term variability, which provides a dynamic insight into sympathovagal balance. Although spectral indices are largely approved in clinical practice for their prognostic value in cardiovascular disease, they are not fully informative of autonomic function. Mechanisms in cardiovascular control may overlap at the same frequency (Japundžić-Žigon, 1998), for instance sympathetic activity and slow respiration in humans, but also fast sympathetic frequencies (above 1 Hz) vasoconstrict blood vessels and do not produce oscillations due to low-pass filter properties of adrenergic receptors (Julien *et al.* 2001). We employed two stressors that induce different behaviours that govern different cardiovascular responses. Restraint induces active coping behaviour, i.e. struggling, while shaker stress is coupled with a passive coping reaction. Classical escape reaction (fight and flight) involves dorsomedial hypothalamic area (hypothalamic defense area) and inhibition of the baroreflex that allows concomitant increase of BP and HR. Hatton *et al.* (1997) provided direct evidence in BHRs, by baroreceptor deafferentation and pharmacological blockade, that the baroreceptor does not contribute to BP maintenance during air-jet stress exposure. In contrast, the cardiovascular response underlying a passive behavioural response, such as freezing and playing dead reaction, is governed from the dorsolateral hypothalamic area (hypothalamic vigilance area) and is characterized by a slight change in BP and HR (Duan *et al.* 1996) and no inhibition of the baroreflex function. Correspondingly, in our experiments the cardiovascular response of rats to stressors was found to be defined by the stressor and by the rat strain. Acute shaker stress induced a passive coping strategy and did not modify baroreflex sensitivity. The maximal increase of systolic and diastolic BP was greater in BHRs than in normotensive rats, whereas the difference of change in diastolic BP, as well as the increase of HR, was smaller in BHRs, possibly due to enhanced baroreflex sensitivity of BHRs (Table 2 and Fig. 3*A*). Frequency analysis of the HR revealed concomitant sympathetic and vagal activation in normotensive rats during exposure to acute and chronic shaker stress. At the same time, no increase in sympathetic outflow to blood vessels was detected by the LF BP marker, possibly due to the simultaneous release of vasodilating substance(s) locally, which maintains blood flow to muscles involved in balancing against the shaking platform. In BHRs exposed to acute and chronic shaker stress, spectral markers did not indicate any difference in sympathovagal outflow to the heart, pointing to the failure of their autonomic nervous system to cope with stress adequately. This could be due to the absence of baroreflex control in the LF and HF range.

Restraint is the strongest stress inducer in rats (Chen & Herbert, 1995; McDougall *et al.* 2000). In both rat strains, acute restraint stress increased BP, HR and their LF variability, depicting obvious sympathetic stimulation typical for an active coping strategy. While a decrease of baroreflex sensitivity and operating range was noted in normotensive rats, the baroreflex sensitivity of BHRs was preserved and even increased during chronic conditions (Table 3 and Fig. 6). At the same time, LF variability of BP and HR was increased only in BHRs, revealing failure to habituate. Furthermore, the shift of the spectral frequencies in the HR spectra of normotensive rats (LF/HF HR reduction) exposed to acute restraint indicates that the heart is dominantly controlled by the vagus, hence protected against sympathetic overstimulation. This effect was not seen in BHRs, and when they were repeatedly exposed to restraint, reverse frequency distribution in their HR spectrum occurred (LF/HF HR increase), indicating that the heart is dominantly controlled by the sympathetic nervous system. These results give experimental evidence about the imbalance of the autonomic nervous system and vulnerability of BHRs during exposure to acute and chronic environmental stress.

Contrary to our findings in BHRs, clinical studies in borderline hypertensive humans report decreased baroreflex sensitivity in baseline conditions (Takeshita *et al.* 1975; Watkins *et al.* 1996) or no change (Bovy *et al.* 1983). In addition to the well-known problem of extrapolation of rat data to humans, there are no clear guidelines that classify stages of hypertension in rats as there are in humans. Borderline hypertension in humans includes prehypertensive and mildly hypertensive subjects (Takeshita *et al.* 1975; Bovy *et al.* 1983; Saito, 1983; Watkins *et al.* 1996) with systolic BP < 160 mmHg (Mancia *et al.* 2009). Therefore, systolic BP inclusion criteria for subjects in clinical studies was higher than the systolic BP of BHRs used in the present study (SBP < 140 mmHg). Bearing in mind that baroreceptor reflex sensitivity is negatively correlated to resting blood pressure (Saito, 1983), this might provide a possible explanation for different findings. In addition, some clinical studies included subjects with hyperkinetic borderline hypertension that is characterized by increased baseline cardiac sympathetic drive, while BHRs in this study had no increase in baseline HR. Finally, baroreceptor reflex sensitivity evaluated in borderline hypertensive subjects in clinical trials was derived from supine conditions and provides information mainly about the vagal part of the baroreceptor reflex, whereas we evaluated both vagal and sympathetic branches of the baroreceptor reflex.

In conclusion, the results of this study in BHRs in the prehypertensive stage show that maintenance of BP in baseline conditions is associated with increased sensitivity and resetting of the baroreflex. Although increased reflex sensitivity is preserved during exposure to stress, cardiovascular adaptation is not complete. According to spectral markers, the underpinning mechanism of cardiovascular hyper-responsiveness to stress is a shift from vagal towards sympathetic control of the cardiovascular system.

### Perspectives

It is generally accepted that human hypertension arises from an interplay of heritable factors and chronic social stress. This study provides evidence about cardiovascular vulnerability of BHRs to environmental stress, directing future investigation towards the genetic background of this vulnerability in the nuclei integrating behavioural and neurogenic responses to psychosocial stress. Due to the discrepancy in the findings on the baroreflex sensitivity in BHRs and borderline hypertensive humans, it also raises concerns about the adequacy of the BHR model for human hypertension.

## Supporting information

Online supplementarymaterial for this paper can be accessed at Wiley Online Library:

**Figure S1.** Typical experiments in Wistar rats with the corresponding spectra.

**Figure S2.** Typical experiments in BHRs with the corresponding spectra.

As a service to our authors and readers, this journal provides supporting information supplied by the authors. Such materials are peer-reviewed and may be reorganized for online delivery, but are not copy-edited or typeset. Technical support issues arising from supporting information (other than missing files) should be addressed to the authors.
