# Supplementary figures and images for: Autonomic mechanisms underpinning the stress response in borderline hypertensive rats
Source: Exp Physiol. 2011 Mar 18;96(6):574–89. doi: 10.1113/expphysiol.2010.055970 (PMC3272224; doi:10.1113/expphysiol.2010.055970)

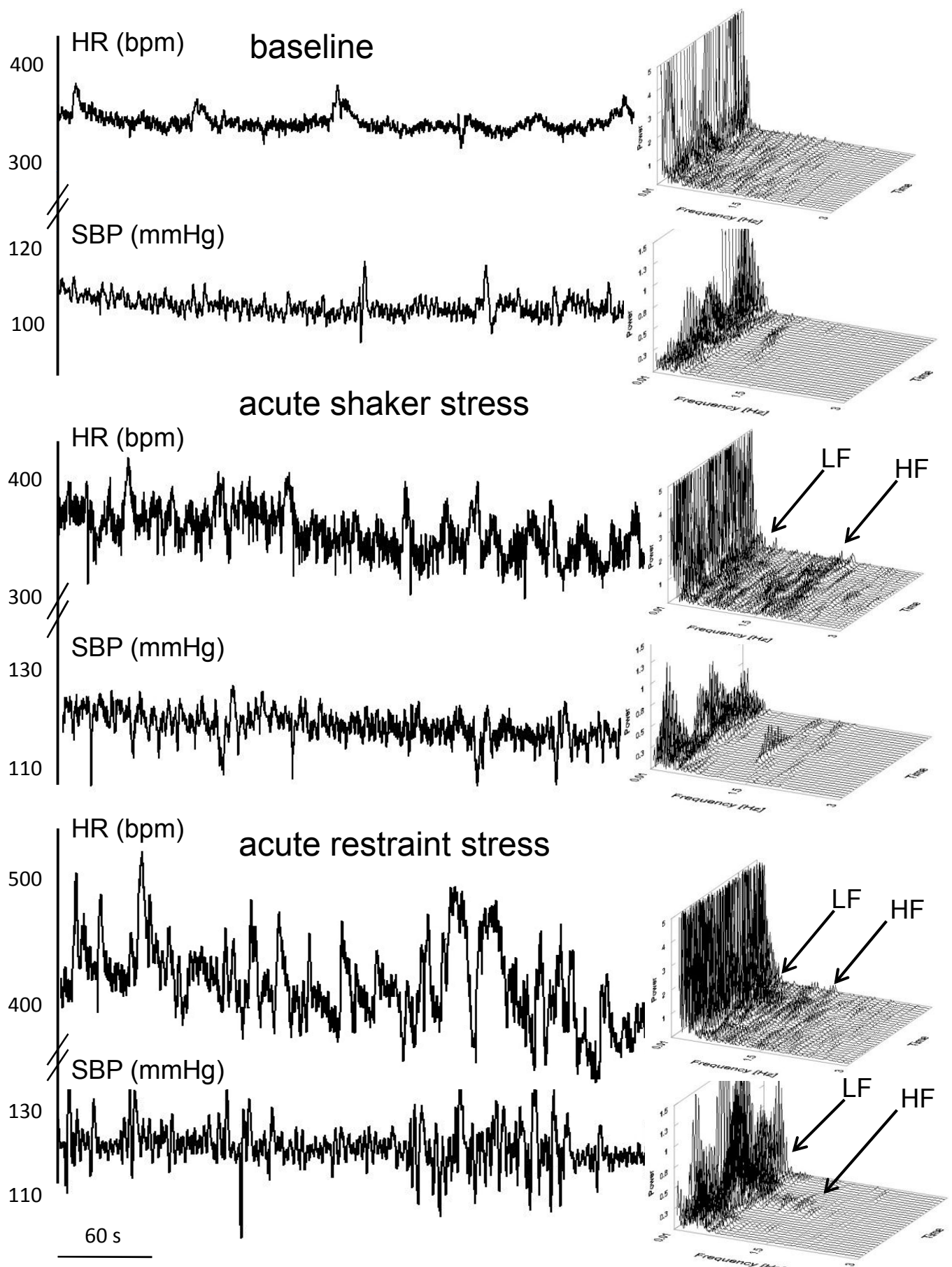

**Supplement figure 1**

Supplement: Supplementary file 1 [file eph0096-0574-SD1.pdf]

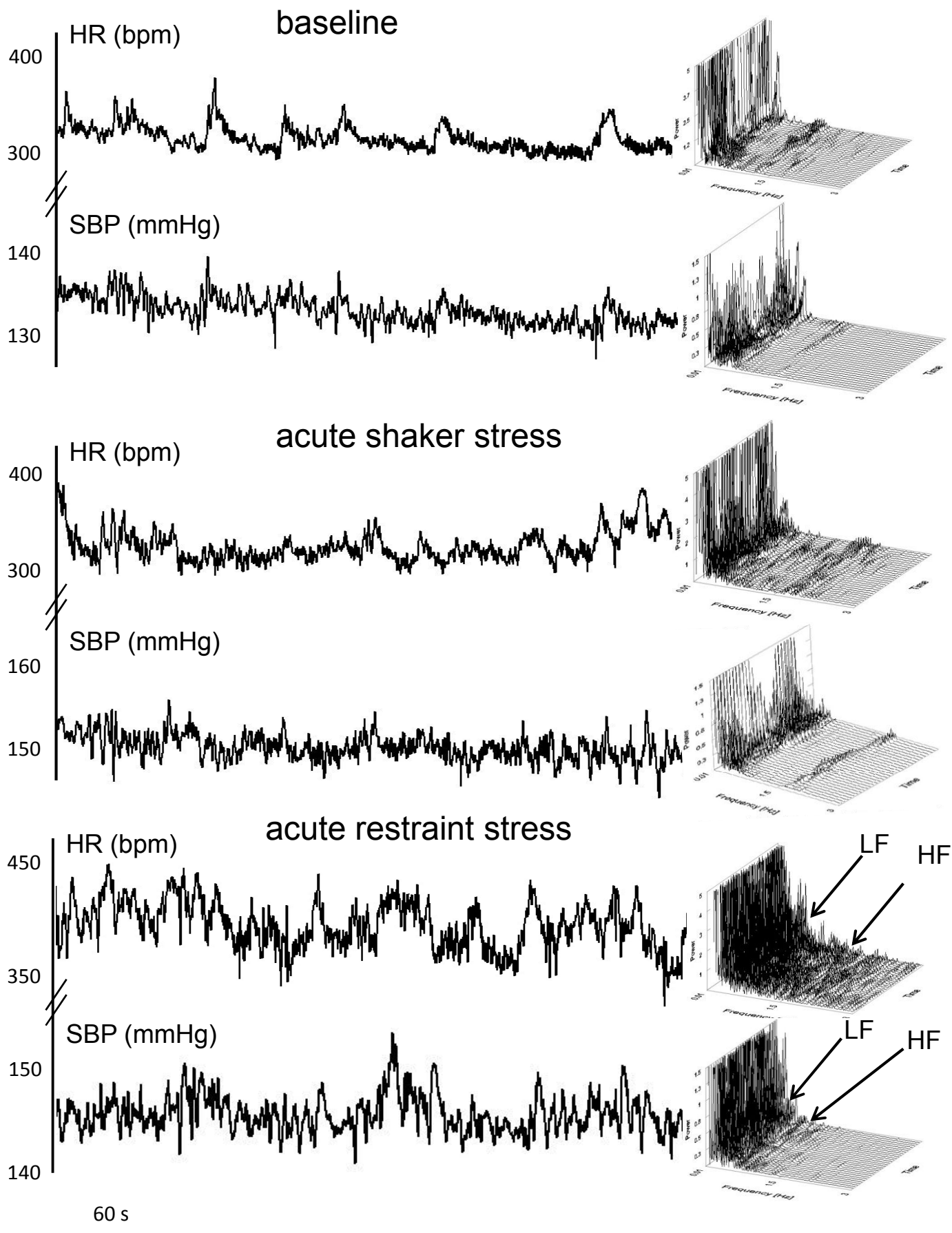

**Supplement figure 2**

Supplement: Supplementary file 2 [file eph0096-0574-SD2.pdf]
